# Experimental data on antibiotic cephalexin removal using hydrogen peroxide and simulated sunlight radiation at lab scale: Effects of pH and H_2_O_2_

**DOI:** 10.1016/j.dib.2020.105437

**Published:** 2020-03-17

**Authors:** Rafael Santiago Cárdenas Sierra, Henry Zúñiga-Benítez, Gustavo A. Peñuela

**Affiliations:** a Grupo GDCON, Facultad de Ingeniería, Sede de Investigación Universitaria (SIU), Universidad de Antioquia UdeA, Calle 70 # 52 -21, Medellín, Colombia; b Departamento de Ingeniería Química, Facultad de Ingeniería, Universidad de Antioquia UdeA, Calle 70 # 52-21, Medellín, Colombia

**Keywords:** Advanced oxidation technologies, Cephalexin, Hydrogen peroxide, Sunlight radiation, Wastewater treatment

## Abstract

Cephalexin (CPX) is a β-lactam antibiotic widely used to treat bacterial infections in the respiratory tract, skin, bones, and ear; a situation that has contributed to its discharge into wastewater (mainly through excretion after ingestion) and its accumulation in water bodies. CPX presence on environmental compartments could interfere in the physiological functions of animals and humans due to the induction of mutagenic and carcinogenic effects.

Different technologies have been evaluated to remove CPX from aqueous matrices. In this way, this work presents the main data regarding the use of the combination of hydrogen peroxide and simulated sunlight radiation in CPX removal. Effects of H_2_O_2_ initial concentration and solution pH were evaluated using a face-centered, central composite design and the response surface methodology. Optimized conditions, under the evaluated experimental range, were established. In addition, data about the total organic carbon and anions content in treated samples were collected.

These data can be useful for the evaluation of the use of H_2_O_2_ and light radiation on organic pollutants removal, the comparison of the effectiveness of different technologies on CPX elimination, and as a starting point to carry out this type of process at pilot or real scale.

Specifications table

**Table:** 

Subject	Chemical Engineering
Specific subject area	Advanced oxidation technologies
Type of data	Table Figure
How data were acquired	Data were obtained by High Performance Liquid Chromatography (HPLC). Solutions Total Organic Carbon (TOC) and anions content (nitrates and sulfates) were determined using the *Standard Methods for the Examination of Water and Wastewater (2017)* methods 526D (High temperature combustion method) and 4110B (Determination of anions by ion chromatography). Statgraphics Centurion XVI software was employed for the statistical analysis of data.
Data format	Raw Analyzed
Parameters for data collection	Data were collected at fixed experimental conditions. Effects of hydrogen peroxide initial concentration and pH on cephalexin removal were evaluated.
Description of data collection	All experimental data were obtained at lab-scale. Optimized conditions, under evaluated experimental range, were established using the Statgraphics Centurion XVI software.
Data source location	Grupo Diagnóstico y Control de la Contaminación (GDCON), Engineering College, Universidad de Antioquia (UdeA), Medellín-Colombia.
Data accessibility	Data are available only in this article

## Value of the data

• Data show that UV/hydrogen peroxide is an appropriate technique to remove antibiotic cephalexin from water.
• Data could benefit researches and institutions working on wastewater treatment, organic pollutants elimination, and advanced oxidation technologies application.
• Data can be employed for the evaluation of the potential use of H_2_O_2_ and light radiation on organic pollutants removal, and as a starting point to carry out this type of process at pilot or real scale.
• Data present optimized conditions that allow to increase CPX removal using H_2_O_2_ and sunlight.
• Data include information regarding the variation of the total organic carbon, nitrates and sulphates of treated samples.
• Data may be useful in future research on antibiotics removal from aquatic environments.

## 1 Data description

Data presented in this work describes the cephalexin removal by the combination of H_2_O_2_ and simulated sunlight. UV/H_2_O_2_ technology consists in the H_2_O_2_ decomposition under UV light presence to generate hydroxyl radicals (OH•) according to Eq. (1) [1,2]. OH• is able to oxidize organic pollutants [3].

(1) $$H_{2}O_{2}+\text{hv}\to 2\;\text{OH}•$$

The reaction kinetics of the UV/H_2_O_2_ process is a function of the solution pH, the peroxide initial concentration and the physicochemical properties of the target pollutant [4]. In addition, as part of the solar radiation has a wavelength corresponding to the UV range, there is a possibility of implementing photo-treatment systems using solar light and H_2_O_2_ for water treatment [5].

The potential application of H_2_O_2_ and simulated sunlight was evaluated considering a face-centered central composite experimental design and the response surface methodology (response: pollutant removal after 30 min of reaction). Table 1 shows the factors and levels evaluated on antibiotic removal using simulated sunlight and H_2_O_2_; and Table 2 corresponds to the experimental results obtained after carried out the proposed experimental design. Total number of experiments: 11.

**Table 1:** Factors and levels evaluated on CPX removal using simulated sunlight and H_2_O_2_.

Factor	Low level	Medium level	High level
pH	3.0	6.0	9.0
H_2_O_2_ initial concentration (mg L^−1^)	2.3	4.6	6.9

**Table 2:** Experimental design for CPX removal using simulated sunlight and H_2_O_2_ (pollutant initial concentration 2.0 mg L^−1^, reaction time 30 min, irradiance 500 W m^−2^).

Test	pH	H_2_O_2_ initial concentration (mg L^−1^)	Pollutant removal (%)	Predicted pollutant removal (%)
1	3.0	6.9	91.4	88.7
2	9.0	2.3	10.3	14.7
3	6.0	4.6	14.7	17.6
4	3.0	2.3	83.2	82.5
5	9.0	4.6	32.9	24.7
6	3.0	4.6	83.3	86.3
7	6.0	4.6	18.1	17.6
8	6.0	2.3	15.0	10.7
9	6.0	4.6	16.4	17.6
10	9.0	6.9	31.0	33.3
11	6.0	6.9	23.9	23.1

**Fig. 1:**
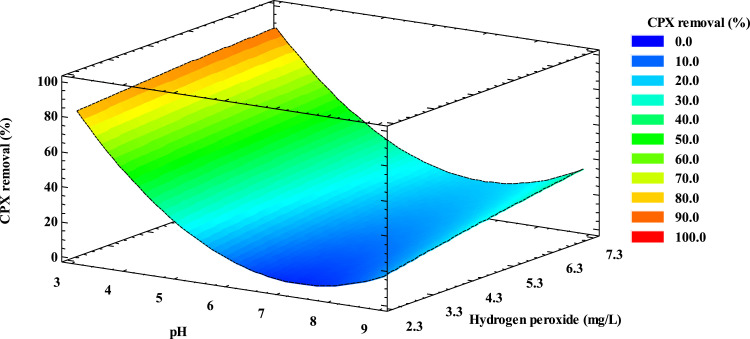
Response surface for CPX removal using simulated sunlight radiation and H_2_O_2_ (pollutant initial concentration 2.0 mg L^−1^, irradiance 500.0 W m^−2^, reaction time 30 min).

Fig. 1 shows the obtained response surface for CPX removal under simulated sunlight radiation and H_2_O_2,_ which allows to predict the pollutant removal under different combinations of pH and H_2_O_2_ initial conditions.

**Fig. 2:**
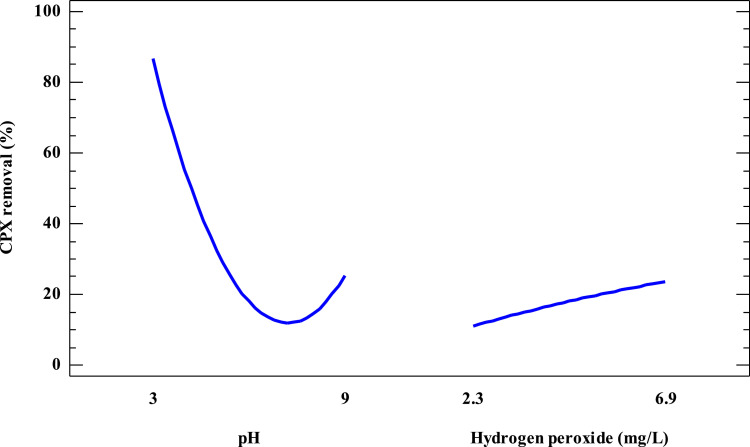
Main effects plot for CPX removal using simulated sunlight radiation and H_2_O_2_ (pollutant initial concentration 2.0 mg L^−1^, irradiance 500.0 W m^−2^, reaction time 30 min).

Fig. 2 corresponds to the associated main effects plot for CPX removal. This figure is useful to determine the effect of each factor on CPX removal without considering the effect of the other variables that intervene in the process.

**Fig. 3:**
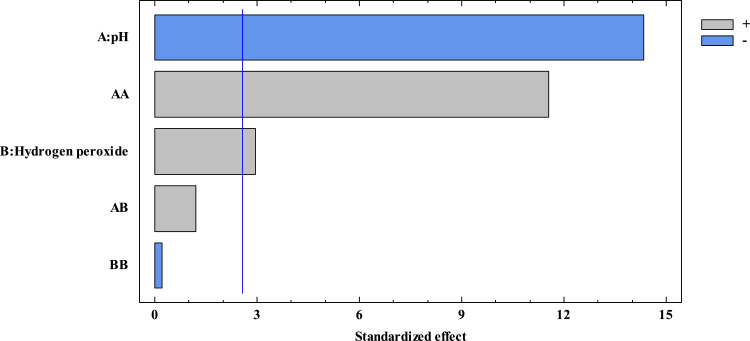
Pareto chart for CPX removal using simulated sunlight radiation and H_2_O_2_ (pollutant initial concentration 2.0 mg L^−1^, irradiance 500.0 W m^−2^, reaction time 30 min).

Fig. 3 is the associated Pareto chart and indicates the magnitude and significance of the effect of each factor and interaction on pollutant elimination.

Statgraphics Centurion XVI software allowed to determine the model (Eq. (2)) that relates the response factor with the evaluated experimental parameters. According to model, conditions that conduct to a higher CPX removal were pH 3.0 and 6.9 mg L^−1^ H_2_O_2_ initial concentration. Coefficient of determination, R^2^, between experimental data and model was 0.986. In addition, Table 2 presents the calculated values regarding CPX removal using the proposed model, which shows that equation predicts CPX elimination, under the studied conditions, adequately.

(2) $$\text{CPX}\;\text{removal}\;\left(\%\right)=227.83-62.85\;\text{pH}+1.28\;H_{2}O_{2}+4.21\;pH^{2}+0.45\;\text{pH}*H_{2}O_{2}-0.14\;H_{2}{O_{2}}^{2}$$

**Fig. 4:**
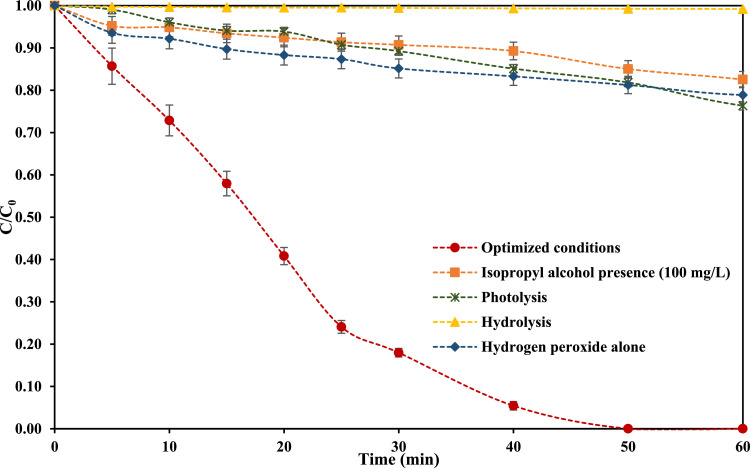
CPX removal under optimized conditions using simulated sunlight radiation and H_2_O_2_ (pollutant initial concentration 2.0 mg L^−1^, irradiance 500.0 W m^−2^, pH 3.0, H_2_O_2_ initial concentration 6.9 mg L^−1^).

Table 3 and Fig. 4 show CPX removal under different experimental conditions including H_2_O_2_/sunlight radiation at optimized conditions, photolysis, hydrolysis (at pH 3.0), oxidation with H_2_O_2_ and the effect of the presence of a scavenger agent (isopropyl alcohol).

**Table 3:** Data regarding CPX removal under optimized conditions using simulated sunlight radiation and H_2_O_2_ (pollutant initial concentration 2.0 mg L^−1^, irradiance 500.0 W m^−2^, pH 3.0, H_2_O_2_ initial concentration 6.9 mg L^−1^).

Sample	Time(min)	C/C_0_				
		Optimized conditions	Photolysis	Hydrolysis	Oxidation with H_2_O_2_	Isopropyl alcohol presence (100 mg L^−1^)
1	0	1.00	1.00	1.00	1.00	1.00
2	5	0.86	0.99	1.00	0.94	0.95
3	10	0.73	0.96	1.00	0.92	0.95
4	15	0.58	0.94	1.00	0.90	0.93
5	20	0.41	0.94	1.00	0.88	0.92
6	25	0.24	0.91	1.00	0.87	0.91
7	30	0.18	0.89	0.99	0.85	0.91
8	40	0.05	0.85	0.99	0.83	0.89
9	50	0.00	0.82	0.99	0.81	0.85
10	60	0.00	0.76	0.99	0.79	0.83

**Fig. 5:**
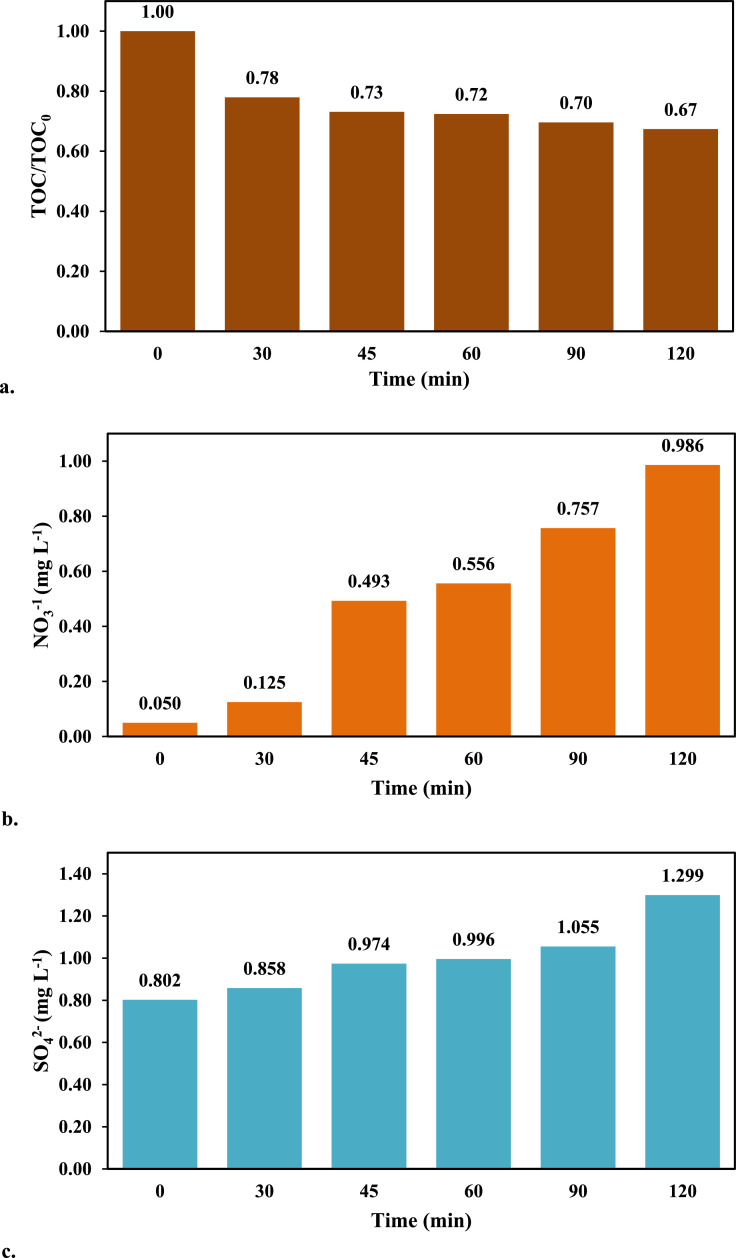
a. Total organic carbon, b. Nitrates and c. sulfate variation during CPX removal under optimized conditions using simulated sunlight radiation.

Finally, Fig. 5 presents information regarding the total organic matter (TOC), nitrates and sulfates content in treated samples.

## 2 Experimental design, materials, and methods

### 2.1 Materials

All the aqueous solutions were prepared using ultra-pure water (Milli-Q water, 18.2 MΩ cm). Cephalexin (98.0%, AK Scientific), H_2_O_2_ (35.0% w/w, Merck) and isopropyl alcohol (99.9%, Merck) were used to carry out the experiments. Control of pH was done using concentrated solutions of NaOH (0.1 N) and HCl (1.0 N) obtained from Alfa-Aesar. Sodium thiosulfate pentahydrate (Na_2_S_2_O_3_•5H_2_O, Sigma Aldrich) was employed for quenching remaining H_2_O_2_ after sampling process; and acetonitrile for chromatographic analysis was of LC/MS grade.

### 2.2 Photocatalytic system

Pyrex flasks containing 50.0 mL of solution (CPX initial concentration 2.0 mg L^−1^) were used for photo-treatment. Solutions pH was adjusted after the additon of CPX and H_2_O_2_. Experiments were carried out using a Suntest CPS+ (Atlas) photosimulator equipped with a xenon lamp that delivered ligth with a spectrum similar to that of the sun. Irradiance during experiments was 500 W m^−2^. Distance from the lamp to the liquid surface was ∼20.0 cm, and the liquid depth inside the flasks was ∼5.0 cm.

### 2.3 Experimental design

The effects of solution pH and H_2_O_2_ initial concentration were evaluated using a face-centered central composite design, and considering the levels presented by Table 1. The total number of experiments was 11 (three central points). Data were analyzed using the Statgraphics Centurion XVI software with a confidence level of 95.0%.

Optimized conditions were selected having into account the polynomical model stablished, after a non-linear regresion of data, by the statistical software (Eq. (2))

Tests regarding polluntat removal under optimized conditions, hydrolysis, photolysis, oxidation with H_2_O_2_ and effect of an scavenger agent presence were done in triplicate.

### 2.4 Analytical methods

Samples of 0.75 mL were withdrawn at different time intervals during the experiments, then 0.25 mL of Na_2_S_2_O_3_•5H_2_O (100 mg L^−1^) were added to inhibit the potential oxidative effect of remaining H_2_O_2_.

CPX concentration was determined using an Agilent 1200 Series HPLC system, a Kinetex C18 column (silica with 100 Å pore diameter, 2.5 μm, 4.6 × 150 mm), and a diode array detector set to 261.4 nm. A mixture of acetonitrile/water (90:10, v/v) was used as mobile phase (flow rate 0.55 mL min^−1^). injection volume was 80 μL, and column temperature was 35.0 °C.

Total Organic Carbon (TOC), nitrates and sulfates were determined using an APOLLO 9000 Combustion TOC Analyzer (Teledyne Tekmar) and a Dionex Integrion HPIC system (Thermo Scientific) respectively. *Standard Methods for the Examination of Water and Wastewater (2017)* [6] methods 526D (High temperature combustion method) and 4110B (Determination of anions by ion chromatography) were also employed.
